# The cytotoxic synergy of nanosecond electric pulses and low temperature leads to apoptosis

**DOI:** 10.1038/srep36835

**Published:** 2016-11-11

**Authors:** Claudia Muratori, Andrei G. Pakhomov, Elena C. Gianulis, Sarah Damsbo Jensen, Olga N. Pakhomova

**Affiliations:** 1 Frank Reidy Research Center for Bioelectrics, Old Dominion University, Norfolk, VA 23508, USA

## Abstract

Electroporation by nanosecond electric pulses (nsEP) is an emerging modality for tumor ablation. Here we show the efficient induction of apoptosis even by a non-toxic nsEP exposure when it is followed by a 30-min chilling on ice. This chilling itself had no impact on the survival of U-937 or HPAF-II cells, but caused more than 75% lethality in nsEP-treated cells (300 ns, 1.8-7 kV/cm, 50-700 pulses). The cell death was largely delayed by 5-23 hr and was accompanied by a 5-fold activation of caspase 3/7 (compared to nsEP without chilling) and more than 60% cleavage of poly-ADP ribose polymerase (compared to less than 5% in controls or after nsEP or chilling applied separately). When nsEP caused a transient permeabilization of 83% of cells to propidium iodide, cells placed at 37 °C resealed in 10 min, whereas 60% of cells placed on ice remained propidium-permeable even in 30 min. The delayed membrane resealing caused cell swelling, which could be blocked by an isosmotic addition of a pore-impermeable solute (sucrose). However, the block of swelling did not prevent the delayed cell death by apoptosis. The potent enhancement of nsEP cytotoxicity by subsequent non-damaging chilling may find applications in tumor ablation therapies.

High amplitude electric pulses of nanosecond duration (nsEP) have been recently proposed as a new local and minimally invasive modality to treat tumors. Advantages of nsEP over other ablation methods include preservation of the extracellular matrix and reduced collateral damage to healthy tissue; relative simplicity of the treatment; and fast recovery.

The cytoxicity of nsEP has been demonstrated in multiple cancer cell types *in vitro*^1^,^2^,^3^,^4^,^5^,^6^. In early studies, the massive externalization of phosphatidylserine (PS) in response to nsEP was interpreted as a sign of apoptosis^7^,^8^,^9^, and apoptosis was considered the prevalent mechanism of cell death induced by nsEP. Indeed, several groups have shown apoptosis in nsEP-treated cells using different apoptotic hallmarks such as activation of caspase, DNA fragmentation, cytochrome *C* release in the cytoplasm, and poly-ADP ribose polymerase (PARP) cleavage^6^,^8^,^10^,^11^. However, later studies revealed that nsEP open pores in the plasma membrane^12^,^13^,^14^,^15^ and cause an increase in intracellular calcium concentration, thus inducing scramblase activation and PS externalization^16^,^17^. Moreover, nsEP-induced PS externalization occurs within seconds after exposure, which is too fast for an organized apoptotic process^12^,^18^,^19^,^20^. In view of these data, the use of PS externalization as a sign for induction of apoptosis by nsEP has become debatable.

More recently, several groups including ours reported that cells exposed to nsEP swell and may die because of necrosis within the first several hours after the treatment^5^,^6^,^10^,^21^,^22^,^23^. Necrosis is caused by the presence of long lived nanopores and colloid-osmotic imbalance which leads to cell swelling and membrane rupture. Alternatively, nsEP can evoke osmotically-independent, delayed necrotic death mediated by an abrupt and Ca^2+^-dependent expansion of plasma membrane pores^24^.

While the induction of apoptosis occurs in response to nsEP, has been documented beyond doubt, the balance of apoptotic and necrotic processes, and how this equilibrium is influenced by the exposure parameters, remain poorly understood.

Despite this incomplete knowledge, nsEP have already been successfully used for cancer ablation in animal models and in human trials^21^,^25^,^26^,^27^,^28^. For instance, 300 ns pulses caused complete remission with no recurrence of murine melanomas in one treatment^28^. In humans, 100 ns pulses caused regression of basal cell carcinoma lesions, with no scarring and no significant side effects^27^. One major obstacle to a wider use of nsEP in the clinic is the limited output voltage of the existing pulse generators, which limits the size of the ablation zone thus requiring multiple electrode insertions and exposures when treating bigger tumors.

In the present study we show that the cytotoxicity of nsEP can be greatly increased by a brief cooling after exposure to electric pulses. When neither nsEP alone nor cooling alone affected cell survival, their combination triggered apoptosis and culminated in 75% cell loss at 23 hr. The likely cause of this strong synergy was hampered resealing of electroporated cells at lower temperatures, which aggravated the disruption of cell homeostasis. However, the facilitation of the colloid-osmotic swelling played little or no role in the induction of the delayed cell death.

## Materials and Methods

### Cell lines and media

In most of the experiments we used U-937 (human monocyte lymphoma) cells. This cell line was chosen because the response of U-937 to electric pulses has been extensively investigated by several groups in the field including ours^5^,^6^,^20^,^24^,^29^,^30^. U-937 and HPAF-II (human pancreatic adenocarcinoma) cells were obtained from ATCC (Manassas, VA). U-937 grow in suspension and were cultured in RPMI-1640 medium (Sigma-Aldrich, St. Louis, MO). HPAF-II grow in a monolayer and were kept in EMEM medium (ATCC). Both growth media were supplemented with L-glutamine (ATCC), 10% (v/v) fetal bovine serum (Atlanta Biologicals, Norcross, GA), 100 U/ml penicillin and 0.1 mg/ml streptomycin (Mediatech Cellgro, Herdon, VA).

### nsEP exposure methods

Cell samples were exposed to nsEP in 1 mm gap electroporation cuvettes (BioSmith, San Diego, CA) at room temperature.

U-937 cells were resuspended at 1.2 to 5 × 10^6^ cell/ml in fresh RPMI medium. For certain experiments, the medium was supplemented with 25 mM HEPES to maintain the pH 7.4 while outside the incubator. 100-μl samples were loaded in the electroporation cuvettes and subjected to either nsEP or sham exposure.

Trapezoidal pulses of 300 ns duration and 700 V amplitude from an AVTECH AVOZ-D2-B-ODA generator (AVTECH Electrosystems, Ottawa, Ontario, Canada) were delivered to electroporation cuvettes via a 50- to 10-Ohm transition module (AVOZ-D2-T, AVTECH Electrosystems) modified into a cuvette holder. Pulse trains of predetermined duration, at the selected repetition rate of 100 Hz, were triggered externally from a model S8800 stimulator (Grass Instrument Co., Quincy, MA). The pulse amplitude and shape were monitored using a 500 MHz, 5 GS/s TDS 3052B oscilloscope (Tektronix, Wilsonville, OR, USA).

nsEP exposure of HPAF-II cells without detachment from the substrate was accomplished by growing the cells on glass coverslips with an indium tin oxide (ITO) conductive layer, and loading these coverslips in EMEM-filled electroporation cuvettes^24^. The ITO layer was deposited on one side of glass coverslips (#0 thickness, 8 mm diameter) by Diamond Coatings (Halesowen, UK). For better cell adherence, the ITO surface was treated with poly-L-ysine. Cells were seeded at 3 × 10^4^ cells per coverslip and cultured overnight in the growth medium. Cells were exposed to 700 pulses (300 ns, 100 Hz) at 600 V, which generated practically uniform electric field of 1.8 kV/cm at the coverslips surface^31^.

### Post-nsEP treatment protocols

Immediately following nsEP exposure, cuvettes were placed on ice or in a water bath at 37 °C for 30 min. The temperature of the samples in the different settings was measured using a thermocouple thermometer (Pysitemp, Clifton, NJ). The temperature of the samples by the end of 30-min incubation on ice and in the water bath averaged 1.6 and 36.1 °C, respectively.

To block cell swelling we used sucrose, a nanopore-impermeable sugar, which was shown to prevent the osmotic water uptake caused by nsEP^32^. U-937 cells (5 × 10^5^/sample) were exposed to nsEP in complete RPMI medium plus 25 mM HEPES and immediately afterward mixed 7:3 with an isosmotic water solution of sucrose (290 mOsm/kg, 280 mM) to yield the fractional osmolality due to sucrose of 87 mOsm/kg. Samples were moved to the different temperatures for 30 min and then diluted 5X with fresh medium. Parallel controls were diluted the same way, but with an isosmotic meso-erythritol solution instead of sucrose. Meso-erythritol is a small sugar, which does not prevent water uptake and therefore served as a control for the equivalent dilution of the medium^33^.

### Propidium iodide permeability assay

Permeability to propidium iodide (PI) was used to measure the kinetics of plasma membrane resealing after nanoelectroporation. Immediately after nsEP exposure all samples were diluted 1:1 with RPMI and placed at 37 °C in the water bath or on ice. At 0, 10, or 30 min post exposure, 20 μl of each cell sample was mixed with an equal volume of 50 μg/ml PI (Sigma) in PBS and placed at 37 °C for 5 min. Cell samples were loaded into a counting chamber of Cellometer Vision (Nexcelom Bioscience LLC, Lawrence, MA) and both bright field transillumination and fluorescence images were acquired. The cell diameters and PI fluorescence intensity of 300–500 cells per sample were measured from the image and logged using Cellometer software. Images were generated using *Grapher* 11 (Golden Software, Golden, CO).

### Viability assays

After exposure to nsEP, cell survival was measured either every hour for 23 hr, using the luminescence-based metabolic cell viability assay Real Time-Glo MT (Promega Corporation, Madison, WI), or at 23 hr, using a resazurin-based metabolic assay Presto Blue (Life Technologies, Grand Island, NY).

To monitor cell survival over 23 hr, U-937 cells were exposed to nsEP in complete RPMI medium with 25 mM HEPES and then incubated on ice or in the water bath at 37 °C for 30 min. Next, the cells were seeded in triplicates in white-wall 96-well plates, the Real Time-Glo reagent was added, and samples were kept in the incubator with 5% CO_2_ for 1 hr. Plates were then sealed from the sides with parafilm and luminescence was acquired every hour using a Synergy 2 microplate reader set at 37 °C (BioTek, Winooski, VT). The triplicate data were averaged, corrected for the background, and considered as a single experiment.

For the Presto blue assay, immediately following the incubation on ice or at 37 °C in the water bath, the cell samples were moved to a 96-well plate (for U-937 cells) or to a 48-well plate (for the HPAF-II on the ITO-coverslips) and incubated for 22 hr before the addition of the Presto Blue reagent for 1 hr. The plate was read with the Synergy 2 microplate reader, with excitation/emission settings at 530/590 nm.

### Caspase 3/7 activity

Caspase activation was measured at 4.5 hr after nsEP using Caspase -Glo 3/7 assay from Promega Corporation, concurrently with measuring cell viability in the same samples. We first recorded fluorescence (Presto Blue/viability) and then added the Caspase -Glo 3/7 assay according to manufacturer’s instructions. Briefly, after the post-nsEP treatments, U-937 cells were plated and incubated at 37 °C in 5% CO_2_ humidified air. The Presto Blue reagent was added 1 hr before measurement. Finally, cell samples were lysed with the Caspase-Glo 3/7 reagent and incubated at room temperature for 1 hr before recording the luminescence signal. As a positive control for apoptosis induction, U-937 were treated with 10 μm staurosporine for 4.5 hr. All conditions were done in triplicates, the data were averaged, corrected for the background, and considered as a single experiment.

### Western blot and quantification of Poly-ADP Ribosome Polymerase (PARP) cleavage

Cleavage of PARP-1 in fragments of 89 and 24 kDa is an established hallmark of apoptosis^34^,^35^. This cleavage is executed by caspases 3 and 7, proteases activated during apoptosis. Both the full-length 116 kDa PARP and its 89 kDa fragment can be detected together by immunoblotting allowing for the quantitation of the apoptotic fraction of cells from the relative amounts of intact and cleaved PARP.

The Western blot procedure was described in detail previously^5^. At 4.5 hr after nsEP treatment, 5 × 10^5^ cells per sample were lysed in a buffer containing 20 mM HEPES (pH 7.5), 200 mM NaCl, 10 mM EDTA, 1% Triton X-100, supplemented with the SIGMAFAST cocktail of protease inhibitors (Sigma). Proteins in the lysate were separated by electrophoresis on a NuPAGE 4–12% Bis-Tris SDS-polyacrylamide gel (Life Technologies) and then transferred to Immune-Blot Low Fluorescence PVDF membrane (Bio-Rad Laboratories, Hercules, CA). The membranes were blocked in the Odyssey blocking buffer for 1 hr at room temperature (LI-COR Biosciences, Lincoln, NE). The primary rabbit anti-PARP polyclonal antibody (Roche Diagnostics GmbH, Mannheim, Germany) was diluted 1∶2,000 in the Odyssey blocker with 0.2% Tween-20. The secondary donkey anti-rabbit IgG (H+L) antibody, conjugated with an infra-red fluorophore IRDye-680LT (LI-COR Biosciences), was diluted 1∶20,000 in the same buffer. The membranes were incubated at room temperature with primary and secondary antibodies for 2 hr and 1 hr, respectively.

The membranes were imaged using Odyssey 9120 Infrared Imaging System (LI-COR Biosciences) in the 700 nm channel. The images were quantified using MetaMorph software (Molecular Devices, Foster City, CA).

The fraction of the cleaved PARP (*K*, %) was calculated as: *K *= 100 × 1.3*S*/ (1.3*S *+ *L*) where *L* and *S* are the fluorescence intensities of the 116 kDa full-length PARP and of the 89 kDa PARP fragment, respectively. The coefficient 1.3 was used for *S* mass correction. As a positive control, apoptosis was induced using 10 μM staurosporine for 4 and 6 hr.

### Statistical analysis

Data are presented as mean +/− SE for *n* independent experiments. Statistical analyses were performed using a two-tailed *t*-test where p < 0.05 was considered statistically significant. Statistical calculations, including data fits, and data plotting were accomplished using Grapher 11 (Golden Software).

## Results

### Post-nsEP cooling induces cell death

**Figure 1:**
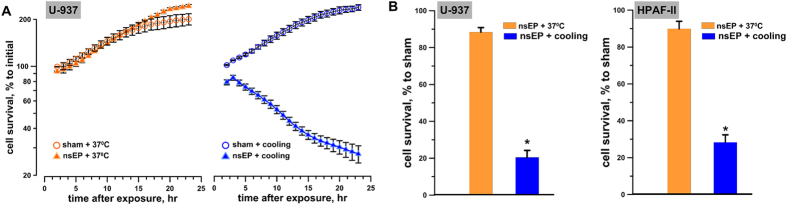
Effect of cooling after nanoelectroporation on survival of different cell types. (**A**) Changes of cell survival in U-937 cells subjected to either sham or nsEP exposure (50 pulses, 300 ns, 7 kV/cm, 100 Hz) followed by a 30 min incubation either at 37 °C or on ice. The survival was monitored from 2 to 23 hr using a Real Time-Glo metabolic assay; the luminescence in “sham+37 °C” group at the earliest timepoint (2 hr) was taken as 100%. Mean +/− s.e for *n *= 3–6. (**B**) Cell survival is profoundly reduced by nsEP+cooling, but not by nsEP alone. The survival was measured at 23 hr after nsEP exposure by the Presto blue assay and expressed in % to sham-exposed parallel control at 23 hr. The nsEP exposure was 50, 300 ns pulses, at 7 kV/cm, 100 Hz for U-937 cells (left panel) and 700, 300 ns pules at 1.8 kV/cm, 100 Hz for HPAF-II cells (right panel). Mean +/− s.e. for n = 6–8, *p < 0.001.

To study the effect of temperature on cell survival after EP exposure, U-937 cells were exposed at room temperature (RT) to 50, 300-ns, 7 kV/cm pulses delivered at 100 Hz. Immediately after the exposure, samples were either placed on ice, or moved into a 37^ ^°C water bath. Parallel sham-exposed samples were incubated at the different temperatures the same way. In 30 min, all cell samples were plated and cell survival was measured every hour (from 2 to 23 hr post exposure) using the luminescence-based metabolic viability assay Real Time-Glo MT (Fig. 1A). Already at 2 hr post exposure the survival of pulsed cells exposed to transient cooling was diminished. It kept declining over time to about 25% of the starting level, whereas the same nsEP treatment alone or the same cooling alone caused no cell death and did not decelerate cell growth.

The synergistic effect between nsEP and cooling was confirmed when using a different cell line, different pulse parameters, a different exposure procedure, and survival assay (Fig. 1B). Cell survival was measured at 23 h post exposure of U-937 cells in suspension (50 pulses, 300 ns, 7 kV/cm, 100 Hz) and of HPAF-II cells on ITO coverslips (700 pulses, 300 ns, 1.8 kV/cm, 100 Hz). In both these cell lines, nsEP exposure alone had little if any effect on cell survival, whereas its combination with cooling caused 70–80% cell loss (p < 0.001).

### Cooling nsEP treated cells blocks membrane resealing and induces cell swelling

A logical explanation for the cytotoxicity of cooling in nsEP-treated cell treatments could be the inhibition of membrane resealing. Prolonging the leaky membrane condition leads to a potentially fatal imbalance in cellular homeostasis. Indeed, a temperature dependence of the resealing process after the conventional electroporation with micro- or millisecond pulses has been documented in several studies^36^,^37^,^38^,^39^,^40^.

**Figure 2:**
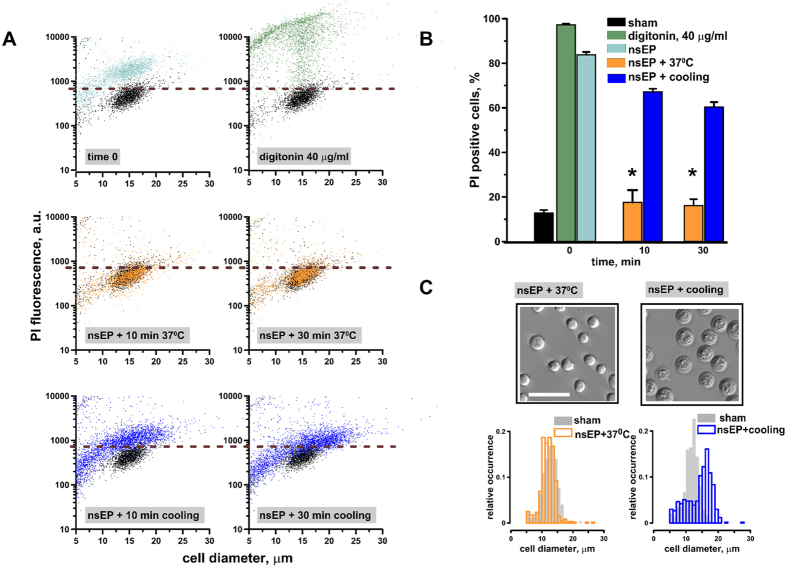
Cooling after nsEP exposure delays membrane resealing causing cell swelling. U-937 cells were exposed to 50, 300 ns pulses (100 Hz, 7 kV/cm) at room temperature, followed by incubation either on ice or at 37 °C. The medium containing PI was added either immediately after nsEP (“time 0”), or after 10 or 30 min of incubation at different temperatures. (**A**) Effect of post-nsEP incubation time and temperature on PI uptake and cell diameter in individual cells. Data for samples not treated with nsEP (sham-exposed negative control) are shown in black in all panels. For a positive control, cells were permeabilized with 40 μg/ml digitonin for 5 min. Horizontal dashed lines show the fluorescence threshold to identify PI-positive cells. (**B**) The fraction of PI-positive cells after different treatments. Mean +/− s.e. for n = 3, *p < 0.001 for the effect of cooling vs 37 °C. (**C**) Post-nsEP cooling for 30 min causes cell swelling (right image and histogram). Filled bars in the histogram show the distribution of cell diameters in sham-exposed control samples. Cells in left panels (nsEP followed 30 min at 37 °C) were not different in appearance or size from the controls. Scale bar: 50 μm. The histogram data are 300–500 cells measured per sample from 3 independent experiments.

The time course of membrane resealing after a nsEP insult (50 pulses, 300 ns, 7 kV/cm, 100 Hz) was assessed by propidium iodide (PI) entry and osmotically-driven swelling in U-937 cells. Immediately after the exposure, 83 +/−1.2% cells were permeable to PI suggesting the opening of PI-permeable pores in the cell plasma membrane (Fig. 2A,B). At 10 min post exposure, cells incubated at 37 °C were already impermeable to PI whereas 67 +/−1.4% of the cells incubated on ice remained permeable (p < 0.001). The effect of cooling became even more prominent at 30 min when cells displayed profound swelling and 60 +/−5.5% of them remained permeable to PI (Fig. 2A–C). Cooling after nsEP exposure caused a drastic morphologic change (Fig. 2C) which resembled what is observed in cells after a hypotonic stress^41^. The reason for the post-nsEP swelling in the isosmotic medium is the presence of the large intracellular solutes, which remain membrane impermeable after nsEP, thereby creating a colloid-osmotic gradient to attract water^42^,^43^,^44^. The modal diameter of nsEP-treated cells incubated on ice increased to 16.5 μm compared to 12.8 μm in cells incubated at 37 °C (Fig. 2C).

### Sucrose inhibits swelling but fails to prevent cell death caused by cooling after nsEP

Several studies reported necrosis due to the colloid-osmotic cell swelling as a predominant mechanism of cell death after exposure to nsEP^5^,^6^,^10^,^21^,^22^,^23^. This mechanism could be blocked by isosmotic addition of a nanopore-impermeable solute (such as sucrose) to the growth medium^32^. Here we employed the same approach to test if the uncontrolled swelling is responsible for death of cells subjected to cooling after nsEP.

Immediately after nsEP exposure (50 pulses, 300 ns, 7 kV/cm, 100 Hz), U-937 cell samples were mixed with a sucrose or meso-erythritol solution to yield the fractional osmolality due to the sugars of 87 mOsm/kg. In contrast to sucrose, smaller meso-erythritol is a pore-permeable solute, which is not expected to prevent swelling^33^; therefore it served as a control for possible effect of the dilution of the growth medium. The samples were kept at 37 °C or on ice for 30 min, then aliquots were collected to assess cell diameters. The remaining volumes were diluted 5X with RPMI medium and cell survival was monitored continuously for 23 hr. Sham-exposed control samples were subjected to the same temperature incubation and media dilutions.

**Figure 3:**
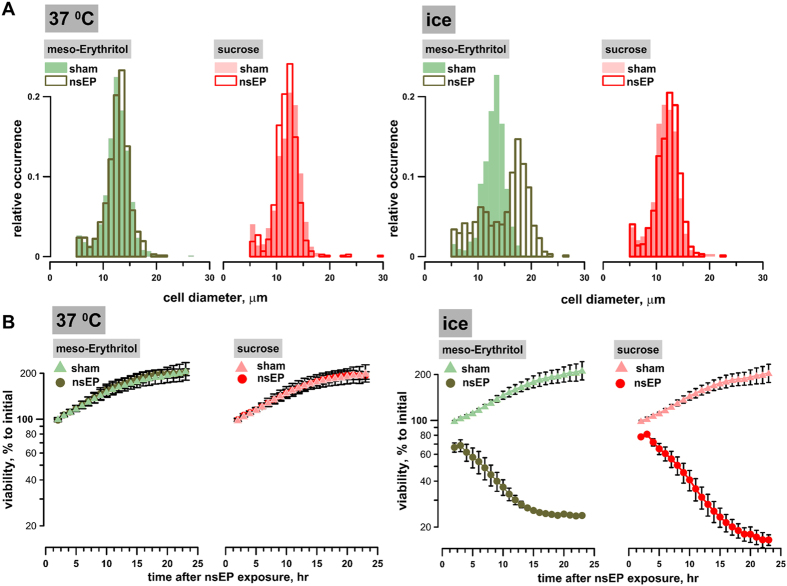
Sucrose inhibits cell swelling (**A**) but does not prevent cell death (**B**) caused by combining nsEP with cooling. U-937 cells were exposed to 50, 300 ns pulses (100 Hz, 7 kV/cm) in the RPMI medium and immediately diluted with isosmotic media containing either meso-erythritol or sucrose; see text for more details. The samples were placed in the water bath at 37 °C or on ice for 30 min, continued by incubation at 37 °C. Parallel sham controls were treated the same way. (**A**) The effect of sugars on cell diameter after nsEP+37 °C (left panel) or nsEP + ice (right panel) (**B**) The effect of sugars on cell survival during 23 hr after nsEP + 37 °C (left panel) or nsEP + ice (right panel). The data were normalized to the luminescence value in “sham+37 °C” group at the earliest time point (2 hr). Mean +/− s.e. n = 3.

Although the dilution of RPMI with sucrose completely prevented cell swelling (Fig. 3A) and improved early cell survival (Fig. 3B; between 2 and 5 hr), it did not prevent the delayed cell loss seen when combining nsEP with cooling. At 23 hr after nsEP exposure and cooling the cell survival was similar in the presence or absence of sucrose (Figs 1A and 3B).

To summarize, the presence of sucrose prevented the osmotic water uptake, cell swelling and early cell death from the membrane rupture after nsEP exposure followed by cooling, but the rescued cells died later on nonetheless.

### Cooling after nsEP exposure induces apoptotic cell death

**Figure 4:**
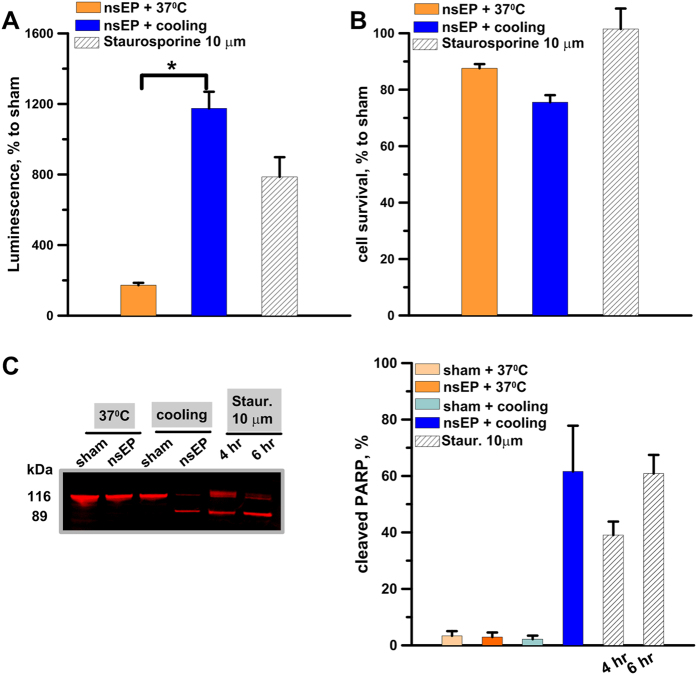
Cooling of nsEP-exposed cells induces caspase 3/7 activation and PARP cleavage. U-937 cells were exposed to 50, 300 ns pulses (100 Hz, 7 kV/cm) and immediately incubated at 37 °C, or placed on ice for 30 min and then in the incubator. Panels (**A**,**B**) show, respectively, the activity of caspase 3/7 and cell survival at 4.5 hr after nsEP with or without cooling. For a positive control, apoptosis was induced by incubation with 10 μm staurosporine for 4.5 hr. Panel C shows a representative Western blot for intact and cleaved PARP (116 and 89 kDa, respectively) and the quantification of the cleaved fraction at 4.5 hr after nsEP exposure.; For a positive control, apoptosis was induced with 10 μm staurosporine. Mean +/− s.e. n = 6–9 (**A**,**B**) or n = 3 (**C**). *p < 0.001 for the difference of nsEP + cooling from nsEP + 37 °C.

The prevalence of the cell death delayed by as much as 5–15 hr after nsEP, as well as the lack of protection when cell swelling and membrane rupture were inhibited, suggested nsEP followed by cooling could have triggered apoptosis. Indeed, we documented strong activation of caspase 3/7 and PARP cleavage in U-937 cells after nsEP (50 pulses, 300 ns, 100 Hz, 7 kV/cm) when it was followed by a 30 min cooling (Fig. 4). The activity of caspase 3/7 at 4.5 hr after nsEP was increased 5-fold by cooling, despite the concurrent 25% cell loss; caspase activation could be even more pronounced than in staurosporine-treated positive controls (Fig. 4A,B).

Another employed hallmark of apoptosis, PARP cleavage, is an intrinsically ratiometric assay, which enables to quantify the ratio of apoptotic and non-apoptotic cells. More than 60% of PARP was cleaved at 4.5 hr after nsEP and cooling; the same nsEP exposure without cooling and the same cooling without preceding nsEP exposure had no effect (<10% of cleaved PARP, Fig. 4C). Same as with the previous assay, the efficiency of nsEP+cooling in inducing apoptosis was comparable or slightly higher than the effect of staurosporine.

Overall, these data demonstrate that cooling after a non-lethal nsEP exposure triggers apoptotic death in most cells.

## Discussion

This study is the first to show that a brief cooling after nsEP exposure can profoundly increase the cytotoxic effect by the induction of apoptosis. The combined effect is strong even when neither cooling nor nsEP applied separately diminish cell survival, thus highlighting the strong synergistic effect of the two modalities. Cooling may assist nsEP-based ablation therapies by allowing to lower pulse voltage and number, or to increase the distance between electrodes without losing the ablation efficiency. Lowering the voltage may help to minimize side effects such as pain, involuntary muscle contractions, and the risk of arrhythmia when treatments are done in the proximity of the heart.

Cooling nanoporated cells might also help to overcome the diverse cytotoxic efficiency of nsEP among different cell types^4^,^6^,^20^,^45^. A recent study shows that the LD_50_ varied profoundly across several commonly used cell types, increasing from 51 J/g for Jurkat to 1861 J/g for HeLa cells^20^. These results suggest that the same ablation protocol may kill one type of cancer but prove very inefficient for another type. This difference might be due to many reasons including plasma membrane physiology or composition, and different abilities to repair nsEP-induced damage.

Restoring the plasma membrane barrier function is mandatory for the cell to survive electroporation. Interestingly, temperature has been shown to affect the cell membrane resealing. Indeed, in this condition nsEP triggered mostly apoptotic cell death.

Our data show that cooling nsEP-treated cells blocks membrane resealing and induces massive cell swelling. This result is consistent with earlier findings using conventional electroporation^36^,^37^,^38^,^39^,^40^; for example, Kinosita and Tsong showed that at 3 °C the permeabilized state of electroporated erythrocytes can be maintained for 20 hr^40^. While it was most logical to expect that the loss of cell volume control leads to necrosis (by swelling culminating in membrane rupture), our experiments showed that it was not the case. In addition to the necrotic cell death seen at about 2 hr post exposure, cooling pulsed cells caused a gradual cell loss that reached maximum at 23 hr after treatment. The long term cell death correlated with a strong activation of caspases and cleavage of PARP denoting the activation of the apoptotic cell death pathway.

The relatively low level of cell death seen at 2 hr after exposure suggests that, once placed in the incubator, cells, which underwent nsEP + cooling treatment, reseal and regain control over their size. A critical question is therefore what triggers apoptosis in these cells. Alterations in the homeostasis of several physiological ions have been shown to influence apoptosis. Several studies have documented an increase of intracellular Ca^2+^ concentration during apoptosis^46^,^47^. Both Ca^2+^ release from the endoplasmic reticulum (ER) and Ca^2+^ influx through Ca^2+^ release-activated Ca^2+^ channels have been proposed to activate programmed cell death^48^. The rise of cytosolic Ca^2+^ caused by permeabilization of the plasma membrane or ER is one the best established effects of nsEP^49^,^50^,^51^,^52^,^53^; however, the effect of Ca^2+^ overload by nsEP was an abrupt opening (or expansion) of plasma membrane pores and necrotic death^24^. The role of Ca^2+^ in the induction of apoptosis by nsEP+cooling has yet to be explored.

Also, the efflux of K^+^ from cells was shown to play a pivotal role in apoptosis^54^. Most cells maintain an osmotic balance through the continuous activity of the Na^+^/K^+^ ATPase pump, which creates an intracellular environment high in K^+^ and low in Na^+ ^55. Prolonged K^+^ efflux is a known effect of conventional electroporation^56^ and probably contributes to nsEP effects as well.

As nsEP affect also intracellular membranes, cooling may increase cell death by prolonging the permeabilized state of intracellular organelles. NsEP have recently been shown to permeabilize nuclear envelope^57^ and mithocondria membrane^58^. Mithocondria play a crucial role in apoptotic cell death^59^. The disruption of the mithocondria barrier function causes the release of apoptosis-inducing proteins such as cytochrome c and cooling after electroporation may augment the release.

Various cellular stress responses and cell death modalities are triggered in response to anti-cancer therapy^60^. Among these, apoptosis has been shown to induce immunogenic cell death (ICD), a death pathway, which stimulates anti-cancer immune response^61^,^62^. ICD is characterized by the release of damage-associated molecular proteins, which induce a pro-inflammatory immune response once exposed on the cell surface or secreted. Among them, calreticulin exposed on the surface of dying cancer cells is essential for the immunogenicity of apoptosis^63^,^64^,^65^. It has recently been shown that calreculin translocates to the cell surface in response to nsEP^66^. Thus, apoptosis induced by cooling may cause ICD and potentially stimulate an anti-cancer immune response.

Combining nanoelectroporation with cooling may considerably improve local tumor ablative therapies. Therefore, future work will aim at demonstrating the benefit of this treatment for tumor ablation *in vivo*.

## Additional Information

**How to cite this article**: Muratori, C. *et al*. The cytotoxic synergy of nanosecond electric pulses and low temperature leads to apoptosis. *Sci. Rep*. **6**, 36835; doi: 10.1038/srep36835 (2016).

**Publisher’s note**: Springer Nature remains neutral with regard to jurisdictional claims in published maps and institutional affiliations.
